# Supplementary material for: Turning microplastics into nanoplastics through digestive fragmentation by Antarctic krill
Source: Nat Commun. 2018 Mar 8;9:1001. doi: 10.1038/s41467-018-03465-9 (PMC5843626; doi:10.1038/s41467-018-03465-9)
Supplement: Supplementary file 1 — Supplementary Information [file 41467_2018_3465_MOESM1_ESM.pdf]

Supplementary Information for

Turning Microplastics into Nanoplastics through Digestive Fragmentation by Antarctic krill

Amanda Dawson* *et al.*

Supplementary Note 1

Microplastic Characterization

According to the manufactures specifications, the fluorescent colouring is embedded in polymer during manufacturing, rather than a fluorescent coating they may chip or peel off the bead, resulting in minimal leaching of colourant (Cospheric LLC CA, USA). Larger particles isolated from krill and faecal pellets were easily recognisable as fragments of whole beads under bright field microscopy (Supplementary Fig. 5). Several randomly selected beads and fragments were imaged using scanning electron microscopy to further confirm the fragments not were simply colourant that had leached from the beads after ingestion (Supplementary Fig. 6).

The manufacturer specifies that >90% of beads are within the specified size range (27-32µm). The mean bead size found on the filter using FIJI[1] was 31.49µm (± 7.621 std. deviation), which was within the range specified by the manufacturer. In cases where the imaging software was unable to distinguish between two or more beads, the agglomeration was counted as single bead. Therefore, size exclusions were applied. The practical size limit of the largest bead was defined as having a diameter of 50µm. This was selected by eye based on the particle size distribution, and was assumed as 2 times the diameter of the smallest whole beads identified (25 µm). This allowed for a conservative approach to be taken, which accounted for beads slightly larger than the manufacturer's specifications, and for any overestimation of the bead diameter by FIJI, due to the use of the fluorescence detection method. The results generated by the microscopic methods used in this study were limited by fluorescence detection by the microscope. Using a laser scanning confocal microscope we were able to achieve relatively high resolution of 1.66µm per pixel. The smallest particle size detected by image analysis on the filters was 1.9µm diameter, which suggests using fluorescence detection could lead to a slight over estimation of size. Using a 10x objective, 1.9µm is defined as the practical limit of detection. However we cannot eliminate the possibility of particles larger than 0.4µm (the pore size of the filter) but smaller than 1.9µm emitting detectable fluorescence, and counted as a particle with a diameter of 1.9µm.

Mortality

One krill died during the feeding bioassay. One krill died during the extended egestion experiment. There was zero mortality during the 24 hour assay.

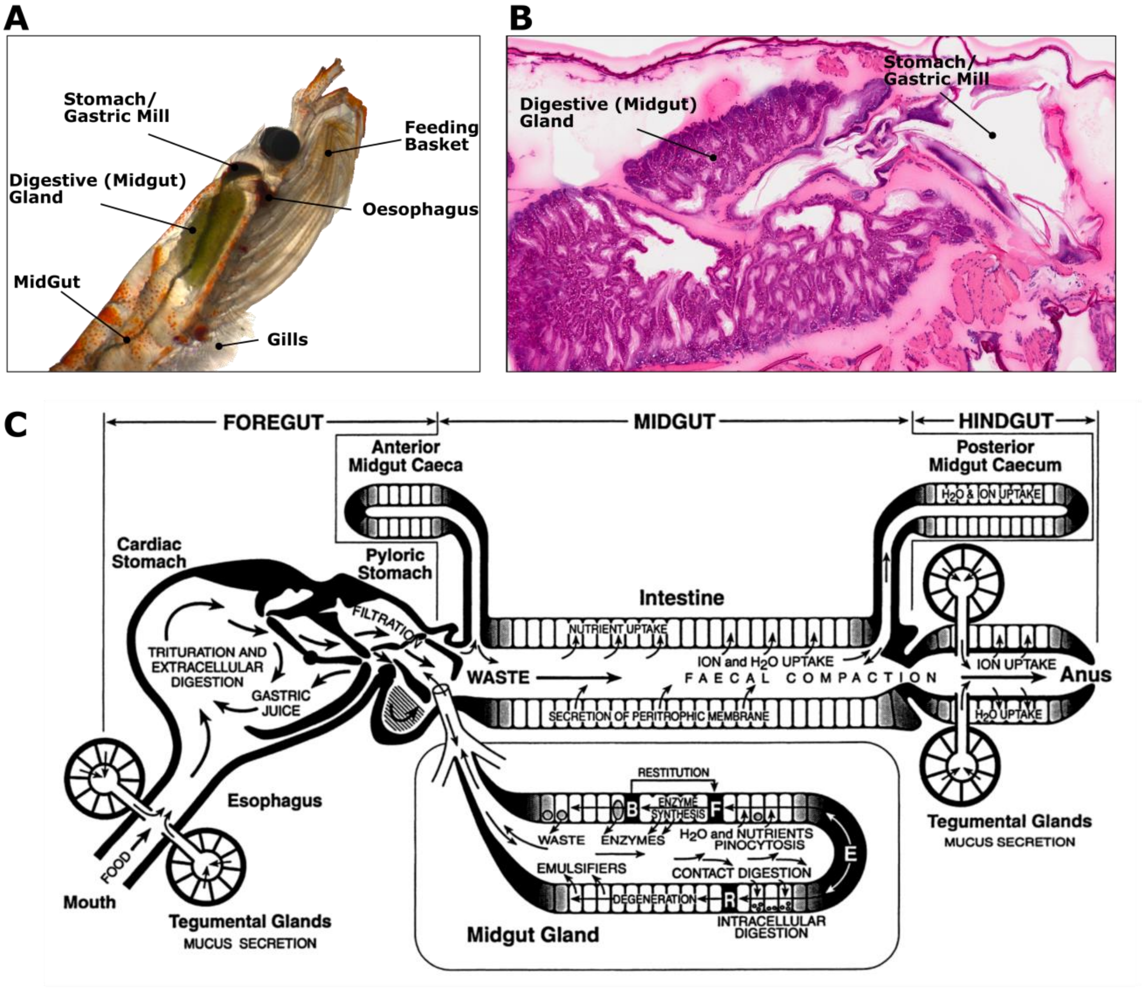

Supplementary Figure 1.

Overview of the digestive system of Antarctic krill (*Euphausia superba*). A) Cephalothorax of *E. superba* showing the location of the digestive organs. The digestive gland is commonly referred to in many texts as the midgut gland; B) Hematoxylin and eosin stained cryosection of the digestive gland and stomach/ gastric mill; C) Diagram of the presumptive digestive tract function of the American lobster (*Homarus americanus*) reproduced from Biology of the Lobster, Douglas E. Conklin, Digestive Physiology and Nutrition, 443., Copyright (1995), with permission from Elsevier. [2] Antarctic krill are presumed to have a similar digestive tract and function. In this article, Cardiac and Pyloric stomach are referred to as Stomach/ Gastric mill, Intestine is referred to as Midgut, and Midgut gland is referred to as Digestive gland

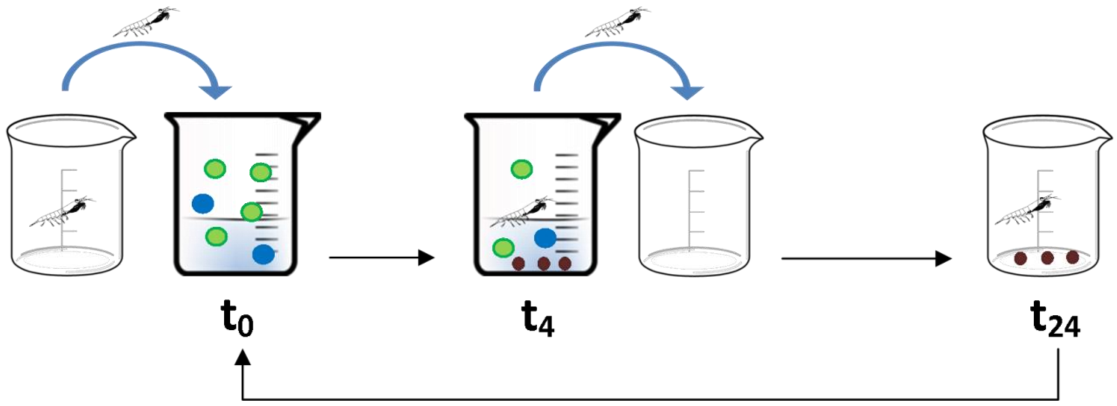

Supplementary Figure 2.

A visual schematic of the exposure design for Antarctic krill (*Euphausia superba*) experiments. Krill were acclimated for 24 hours prior to exposure to microbeads (green circles) and algae (blue circles). T_n indicates hours of exposure. Exposure design was repeated daily for the duration of each experiment. Faecal pellets (brown circles) were only collected in days 1 and 4 of the Particle Size Bioassay, and days 11-15 of the Depuration Bioassay

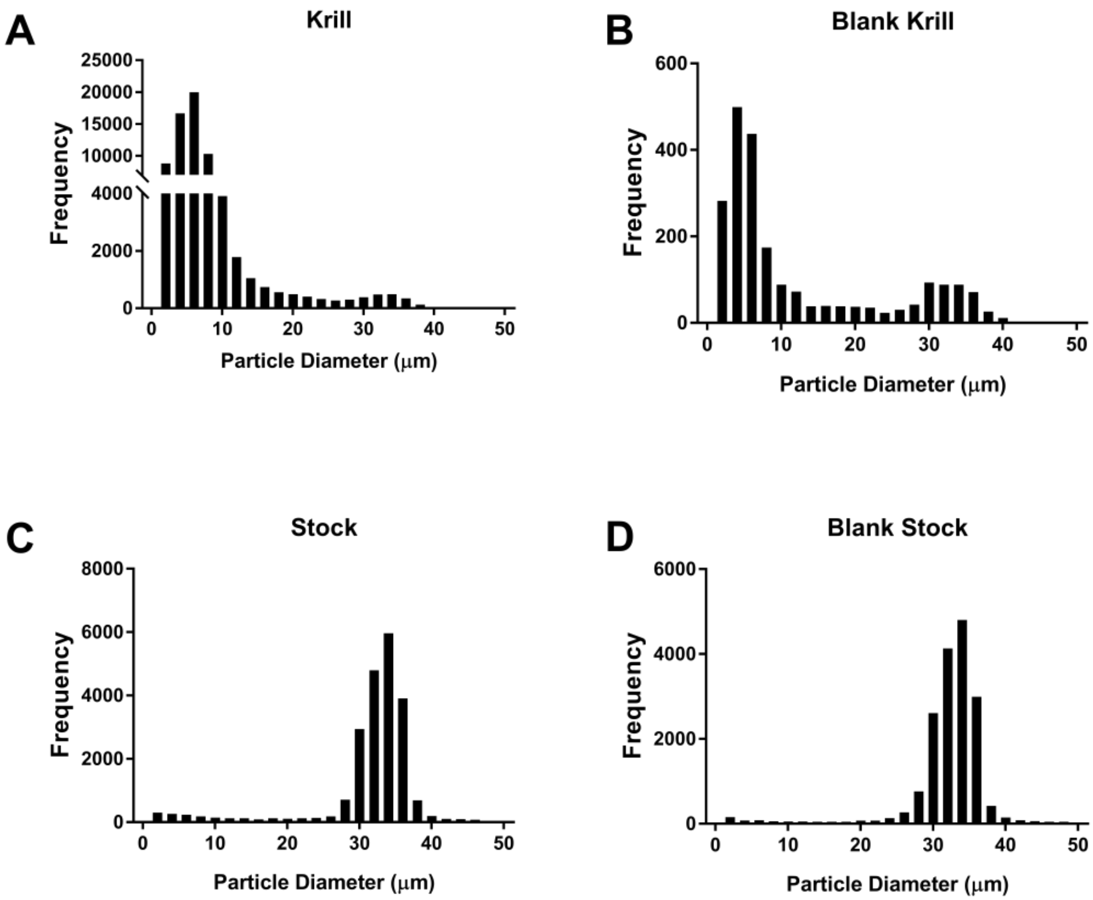

Supplementary Figure 3.

Size distribution of particles isolated from: A) sample Antarctic krill (*Euphausia superba*), B) unhomogenised and enzyme digested Antarctic krill, C) stock suspension, and D) stock suspension enzyme digested. Particles between 25 and 50 µm indicate a whole bead; values below this range are fragments.

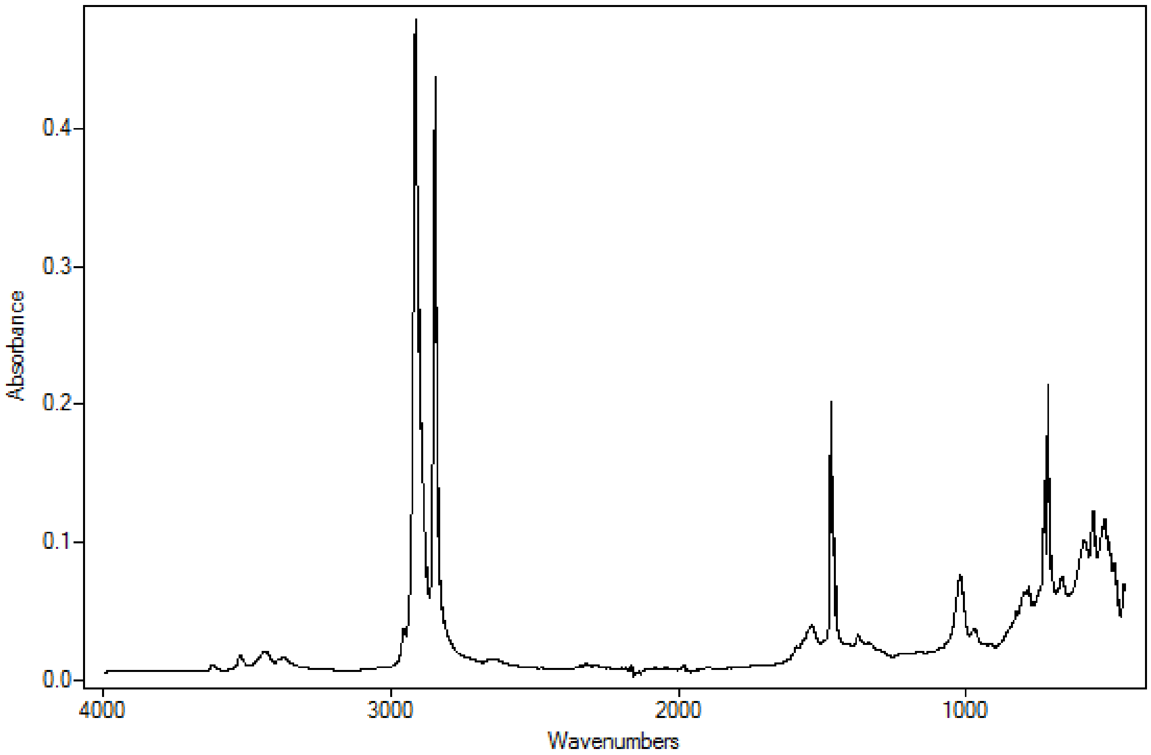

Supplementary Figure 4.

Spectra of microbeads used for exposure in this experiment confirmed to be polyethylene by FTIR spectroscopy using a PerkinElmer FTIR spectrometer. Beads were purchased from Cospheric LLC CA, USA (Product ID - UVPMS-BG-1.025)

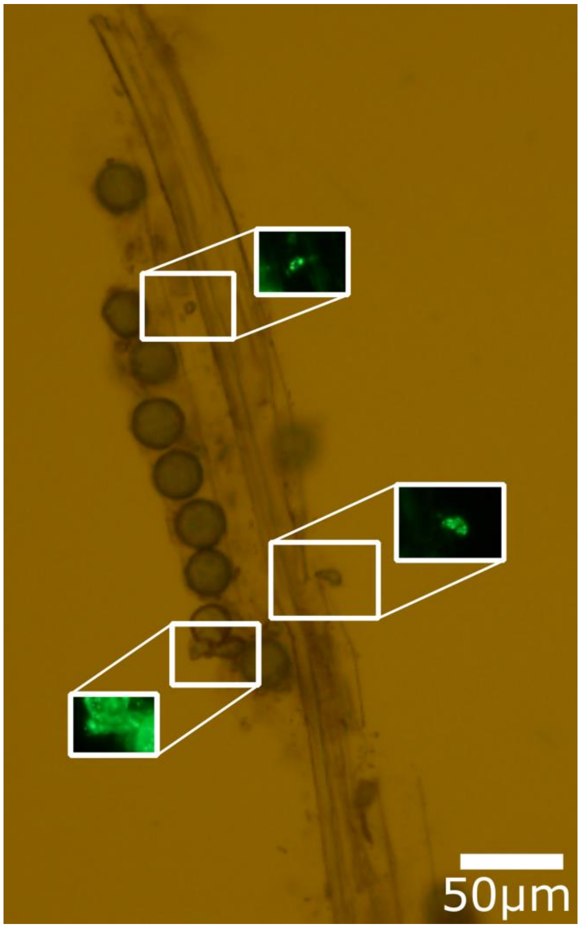

Supplementary Figure 5.

A whole faecal pellet containing whole beads and fragments imaged under bright field and fluorescence microscopy

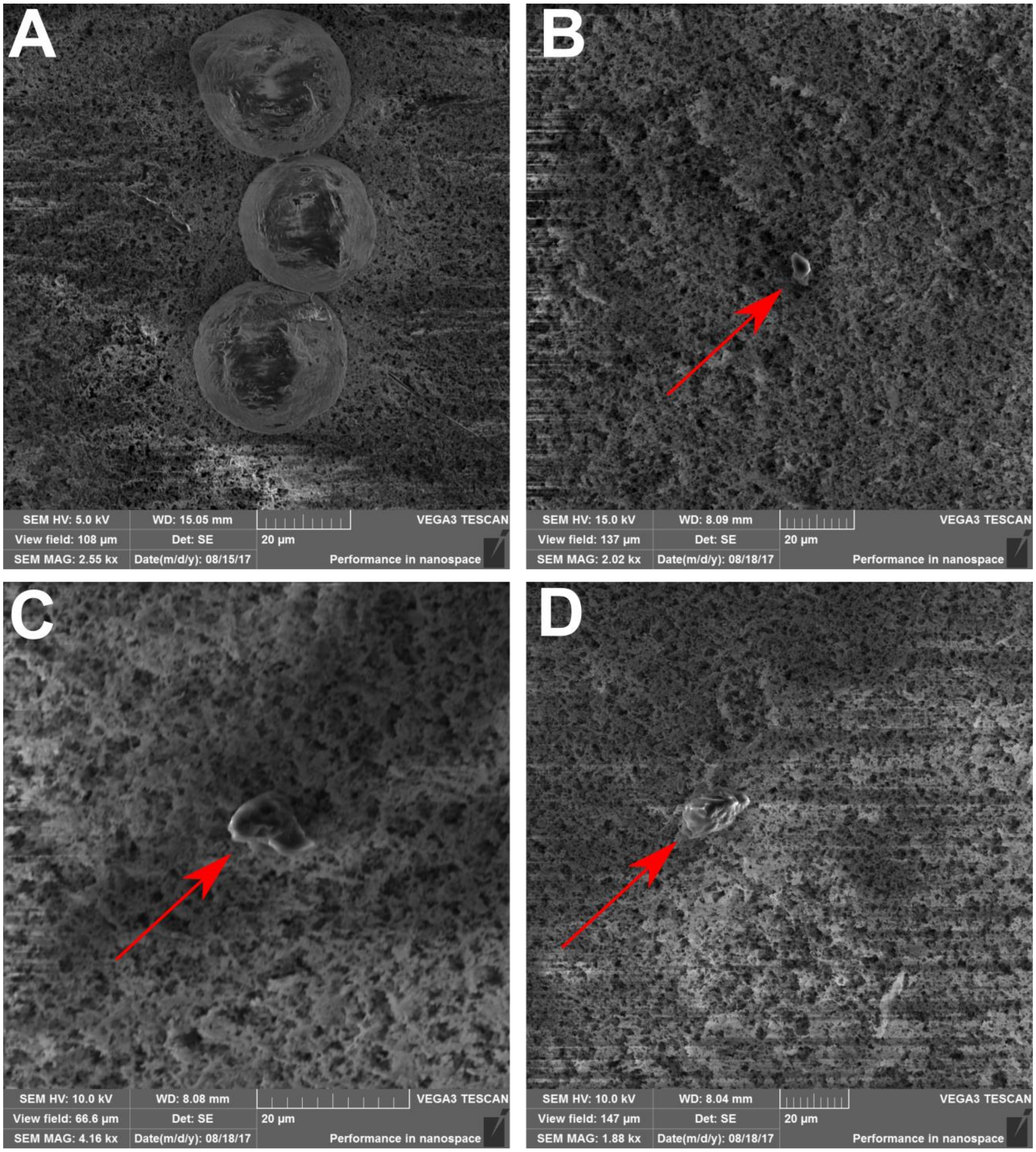

Supplementary Figure 6.

Scanning electron micrograph of ingested beads and fragments filtered onto cellulose filter paper taken using a Vega3 TESCAN Scanning electron microscope. A) Three whole beads, B), C) and D) Bead fragments of various sizes. Arrows indicate the location of fragments.

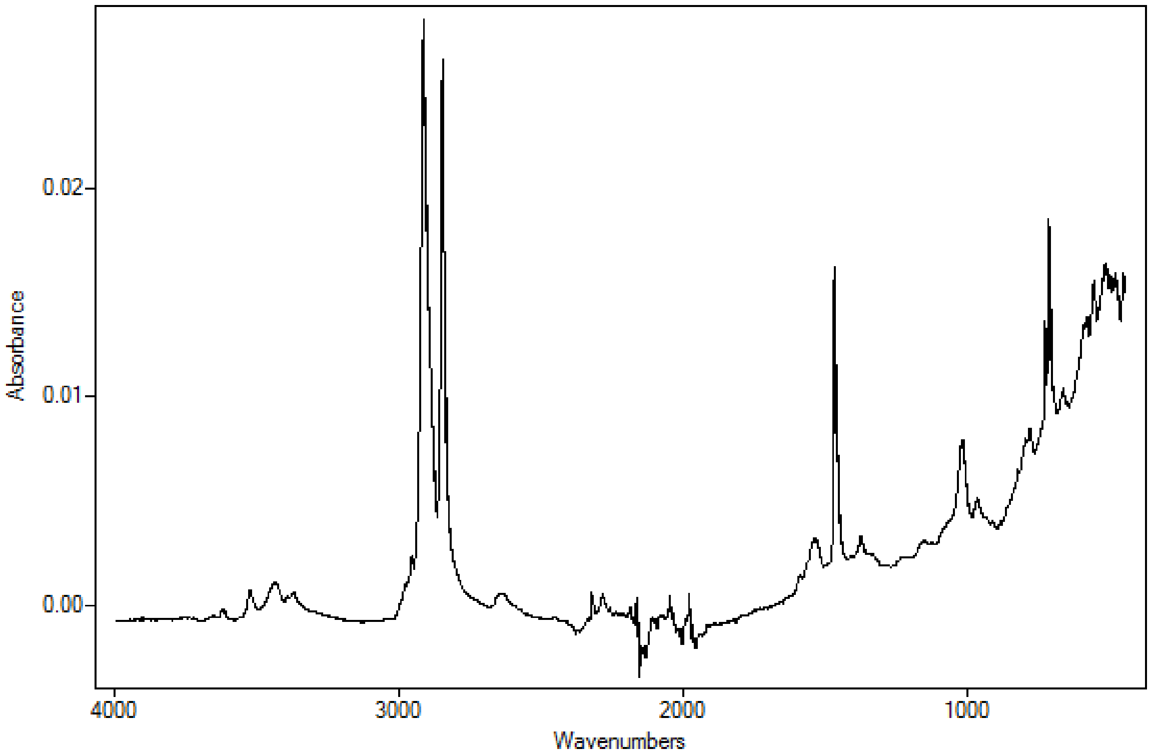

Supplementary Figure 7.

Spectra of bead blanks after enzyme digestion determined by FTIR spectroscopy using a PerkinElmer FTIR spectrometer

Supplementary Table 1.

Seawater parameters for the particle size and tissue localization experiments

	Mean	SD	Min	Max
**Temperature (°C)**	0.55	0.15	0.40	0.80
**pH**	8.15	0.05	8.07	8.21
**Salinity (psu)**	34.98	0.13	34.90	35.20

Supplementary Table 2.

Seawater parameters for the egestion experiments

	Mean	SD	Min	Max
**Temperature (°C)**	-0.04	0.14	-0.30	0.50
**pH**	8.24	0.05	8.13	8.34
**Salinity (psu)**	33.61	0.37	32.98	34.68

Supplementary References

1 Schindelin, J. *et al*. Fiji: an open-source platform for biological-image analysis. *Nat. Methods* **9**, 676-682, doi:10.1038/nmeth.2019 (2012).

2 Conklin, D. E. in *Biology of the Lobster Homarus americanus* (ed Jan Robert Factor) Ch. 16, 441-463 (Academic Press, 1995).
